# Supplementary material for: ProteinShader: illustrative rendering of macromolecules
Source: BMC Struct Biol. 2009 Mar 30;9:19. doi: 10.1186/1472-6807-9-19 (PMC2672931; doi:10.1186/1472-6807-9-19)
Supplement: Additional file 1 — ProteinShader program without source code. This compressed file contains the complete ProteinShader program including associated libraries, but no source code. A README.txt file gives an overview of the ProteinShader distribution, and the index.html file in the help subdirectory has directions on getting started with the program as well as a set of tutorials. [file 1472-6807-9-19-S1.zip › ProteinShader-beta-0_9_4-binary/help/api/org/proteinshader/structure/visitor/class-use/Visitable.html]

Uses of Interface org.proteinshader.structure.visitor.Visitable (ProteinShader API)


|  |  |  |
| --- | --- | --- |
| **Overview** \| **Package** \| **Class** \| **Use** \| **Tree** \| **Deprecated** \| **Index** \| **Help** | |  |
| PREV   NEXT | **FRAMES**    **NO FRAMES**     **All Classes** |  |


---


## **Uses of Interface org.proteinshader.structure.visitor.Visitable**

| Packages that use Visitable | |
| --- | --- |
| **org.proteinshader.structure** | Holds the classes that store information from a Protein Data Bank file: Structure, Model, Chain, AminoAcid, Heterogen, Water, Atom, Bond, Helix, BetaStrand, Loop, *etc*. |

Uses of Visitable in org.proteinshader.structure

| Classes in org.proteinshader.structure that implement Visitable | |
| --- | --- |
| `class` | `AminoAcid`             This concrete subclass of the abstract class Residue stores information on an amino acid in a PDB structure entry. |
| `class` | `Atom`             Extends abstract class Drawable to add data that is unique to an atom (atomID, type, temperature, occupancy, radius, *etc.*). |
| `class` | `BetaStrand`             This concrete subclass of Region holds information specific to a protein BetaStrand. |
| `class` | `Bond`             Extends abstract class Drawable to add data that is unique to a bond between two atoms. |
| `class` | `Chain`             A Chain serves as a container for Residues (AminoAcid, Heterogen, and Water) and Regions (Loop, Helix, and BetaStrand). |
| `class` | `Drawable`             This abstract class defines attributes and methods that are shared by all drawable objects, including the xyz-coordinates for the Drawable's center and methods that will allow each object to be sorted based on distance from the camera. |
| `class` | `Helix`             This concrete subclass of Region holds information specific to a protein Helix. |
| `class` | `Heterogen`             This concrete subclass of the abstract class Residue stores information on a heterogen in a PDB structure entry. |
| `class` | `Loop`             This concrete subclass of Region holds information on the general "loop" regions found between well-defined regions of secondary structure (helices and beta-strands). |
| `class` | `Model`             A Model serves as a container for one or more Chains. |
| `class` | `Region`             Abstract class that contains references to a region of a Chain of AminoAcids. |
| `class` | `Residue`             This abstract class serves as a container for one or more Atoms. |
| `class` | `Segment`             When a protein is drawn as a tube-like structure, a Segment is a length of the tube that corresponds to an AminoAcid. |
| `class` | `Structure`             Structure is the top-level container for objects that store information on a protein structure from the Protein Data Bank. |
| `class` | `Water`             This concrete subclass of the abstract class Residue stores information on a water molecule in a PDB structure entry. |

---


|  |  |  |
| --- | --- | --- |
| **Overview** \| **Package** \| **Class** \| **Use** \| **Tree** \| **Deprecated** \| **Index** \| **Help** | |  |
| PREV   NEXT | **FRAMES**    **NO FRAMES**     **All Classes** |  |


---

# *Copyright © 2007-2008*
